# Use of an online gaming tool, the Veterinary DialogueTrainer, for teaching clinical communication skills to bovine veterinary practitioners

**DOI:** 10.3389/fvets.2023.1192598

**Published:** 2023-07-19

**Authors:** Linda Dorrestein, Jolanda Jansen, Tessa Plagis, Caroline Ritter, Geert Vertenten, Herman W. Barkema

**Affiliations:** ^1^Faculty of Veterinary Medicine, University of Calgary, Calgary, AB, Canada; ^2^St. Anna Advies, Beuningen, Netherlands; ^3^Department of Health Management, Atlantic Veterinary College, University of Prince Edward Island, Charlottetown, PE, Canada; ^4^MSD Animal Health, Boxmeer, Netherlands

**Keywords:** communication skills, serious games, veterinarian-farmer communication, communication training method, veterinarian and farmer, herd health consultancy

## Abstract

Effective clinical communication is essential for bovine veterinary practitioners to establish and maintain positive client relationships. When applied properly in herd health consultancy, it increases client satisfaction and adherence to veterinary advice, and improves patient health and welfare. Although communication skills are often taught by providing feedback on simulated conversations, this has limitations, including time constraints, subjective assessments, and cost. The Veterinary DialogueTrainer (VDT) is an online serious game platform using ‘digital role-play’ with avatars, recently developed to enhance and assess learning outcomes, improve use of learned skills, and increase cost-effectiveness of communication training. The objective was to evaluate its suitability and applicability. Finnish (*n* = 24) and Swedish (*n* = 21) bovine veterinarians participated in communication training using VDT for training and assessment. Participants completed the provided scenario at least twice. After playing a bovine health communication simulation, participants received their scores and feedback on selected conversation options. VDT scores measured multiple aspects of communication on a 0–100% scale, based on motivational interviewing methodology and Calgary-Cambridge guidelines. Mean (±SD) number of attempts participants played the scenario was 4.1 (±2.6, Finland) and 3.9 (±1.3, Sweden), with highest total score reached after a mean of 3.5 (±2.1, Finland) and 3.1 (±1.1, Sweden) attempts. On the first attempt, 39 participants (87%) scored <50% of the highest possible score, whereas most (*n* = 34, 76%) achieved a higher score on the second attempt. Mean total score increased from 15 (±14) to 77% (±33) for Finish participants and from 40 (±22.0) to 87% (±19.4) for Swedish participants. The majority (*n* = 33, 73%) of participants reached a score >80% after 4.0 (±1.6, Finland) or 2.8 (±1.0, Sweden) attempts. Net Promoter Score of the training was +89 (Finland), +88 (Finland) and + 83 (Sweden) on a scale from −100 to +100, indicating that most participants were very likely to recommend the training to other veterinarians. Use of VDT increased communication skills scores but whether it will improve communication skills in practice requires further study. We concluded there is a likely benefit of using the VDT in teaching and monitoring veterinary communication competencies and preparing for offline role-plays and real-life conversations in veterinary practice.

## 1. Introduction

Veterinarians are a key information source of dairy farmers and they have substantial impacts on herd health management decisions (1, 2). Historically, dairy veterinarians were only called to farms to treat individual animals. Although individual animal calls remain part of dairy veterinary practice, the modern dairy veterinarian needs to be a well-rounded herd health and production management consultant (3). This paradigm shift has occurred in the last decades (4–6) with studies directed toward understanding farmers’ perspectives and needs (7–9), the role of the veterinarian, and skills needed in herd health and production management (6, 10, 11).

Well-developed communication skills are a core competency vital to improving herd health, as they have the potential to increase farmer satisfaction with herd health consultancy and adherence to veterinary advice (12–14). Regardless, there is room to improve communication competencies of practicing veterinarians, especially because communication skills training was often lacking in their veterinary curriculum (1, 15, 16). However, these competencies are increasingly addressed in the veterinary curriculum and veterinary continuing education (17–19).

Teaching methods vary among teaching institutions, but often include a didactic part, e.g., lectures, problem-based teaching such as case studies, and experiential learning approaches such as role playing in small groups or facilitated one-on-one learning (20, 21). Communication behavior is more likely to be changed through experiential methods, e.g., role-play, by providing opportunities to practice alternative approaches to communication (22, 23). Unfortunately, there is a paucity of opportunities for veterinary communication training in postgraduate education. Developing such a program for practicing veterinarians involves integral challenges, such as time constraints, costs, veterinarians’ doubts regarding value of training (23, 24), and limitations of traditional role-play training, e.g., simulated clients, reluctance of training participants to engage in role-play (25), and difficulties to administer, assess and repeat (26).

Online serious games have potential for improving continuing communication education for veterinary practitioners. In these games, the concept of gaming has been applied to training and education to facilitate an interactive learning environment, engaging learners to improve knowledge or skills (26, 27). Virtual patients (VPs), simulations of patient encounters in an online interactive computer environment, are examples of such serious games in the human medicine context (28, 29). Although VPs increased communication knowledge and clinical reasoning of students in human medicine education (27, 29), VPs are rarely used in communication skills education for veterinary medicine [reviewed by (30)]. Jeuring et al. (31) introduced “Communicate!” a serious game currently used in the communication skills curriculum for veterinary students in The Netherlands (28). Since then, the “Communicate!”-tool has been further developed and has been renamed to Veterinary DialogueTrainer (VDT). So far, the tool has not been used in continuing education for veterinary practitioners.

Objectives of this study were to: (1) present the Veterinary DialogueTrainer (VDT) as an online communication training tool for veterinary practitioners; (2) evaluate whether the VDT improves virtually assessed communication skills scores in bovine practitioners during clinical communication skills training; and (3) explore participants’ willingness to recommend the tool to other veterinarians. Further, to the best of the authors’ knowledge, this is the first study to explore the use of an online virtual patient in the context of training postgraduate veterinarians, offering guidance for future studies. Successful application of the VDT would improve teaching clinical communication in terms of increasing communication skills in veterinary practitioners, cost-effectiveness of training interventions, and preparing for offline role-plays and real-life conversations in veterinary practice.

## 2. Materials and methods

### 2.1. Participants

Data for this study were retrieved during two communication training events focused on bovine veterinarians, one held in Finland (September 24 and 25, 2019) and the other in Sweden (October 28, 2021). In Finland, 51 bovine veterinarians received an online invitation to participate in a communication training on preventative dairy herd health consultancy. This training was organized by ETKO animal health and economy-project (Kuopio, Finland). The Swedish training, hosted at a Swedish Veterinary Congress (Stockholm, Sweden), was attended by 25 bovine practitioners who were present at the conference and signed up on-site. After the training, participating veterinarians were invited by email to participate in the study.

Inclusion criteria for this study were participation in the training events and having completed the simulation on initiating a herd health conversation (see “Training intervention”) at least twice. Consent was obtained from veterinarians that met inclusion criteria. Ethics approval was obtained from the University of Calgary’s Research Ethics board (REB20-0137).

### 2.2. Veterinary DialogueTrainer

DialogueTrainer is an online serious game platform developed to enhance and assess learning outcomes, improve use of learned skills, and increase cost-effectiveness of communication training (28, 31). Communication simulations specifically built and/or selected for a target group can be provided through the platform and accessed online at any time. In a communication simulation, a player engages in a “digital role-play” with an avatar, a digital persona. These simulations can be used for a wide array of activities, e.g., to support teaching of constructs or skills, practice conversation techniques, and assess learning outcomes. Communication simulations are developed using Communicate! software as used by the existing DialogueTrainer platform (28, 31). This software tool was developed at Utrecht University (Utrecht, the Netherlands), to improve communication skills training of students of various study programs.

**Figure 1:**
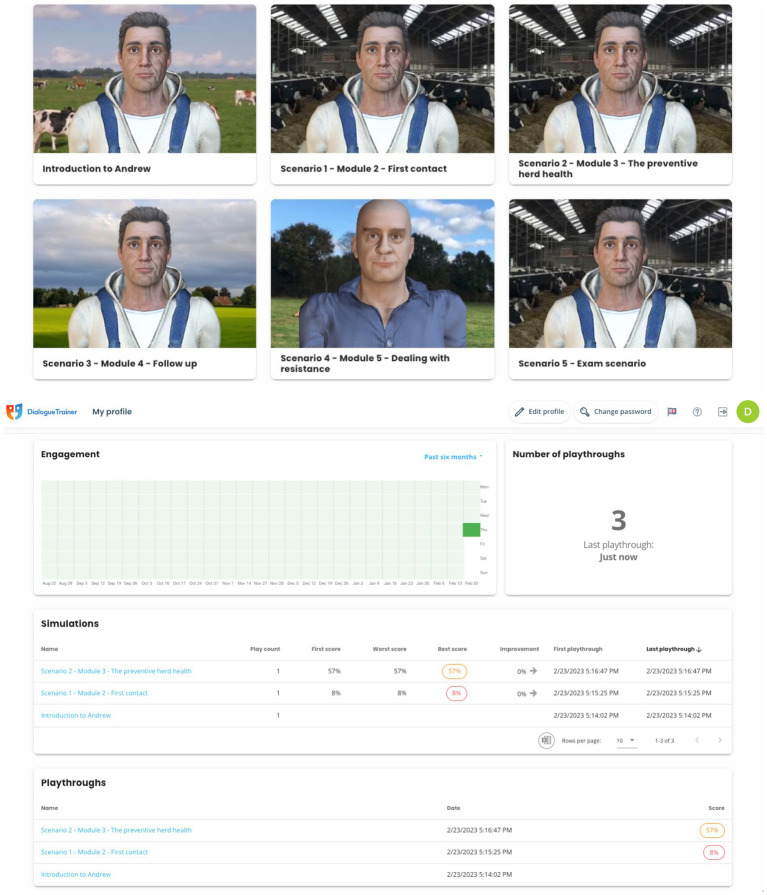
Screenshot of the Veterinary Dialogue Trainer player “dashboard” on which simulated conversations are listed with associated scores.

**Figure 2:**
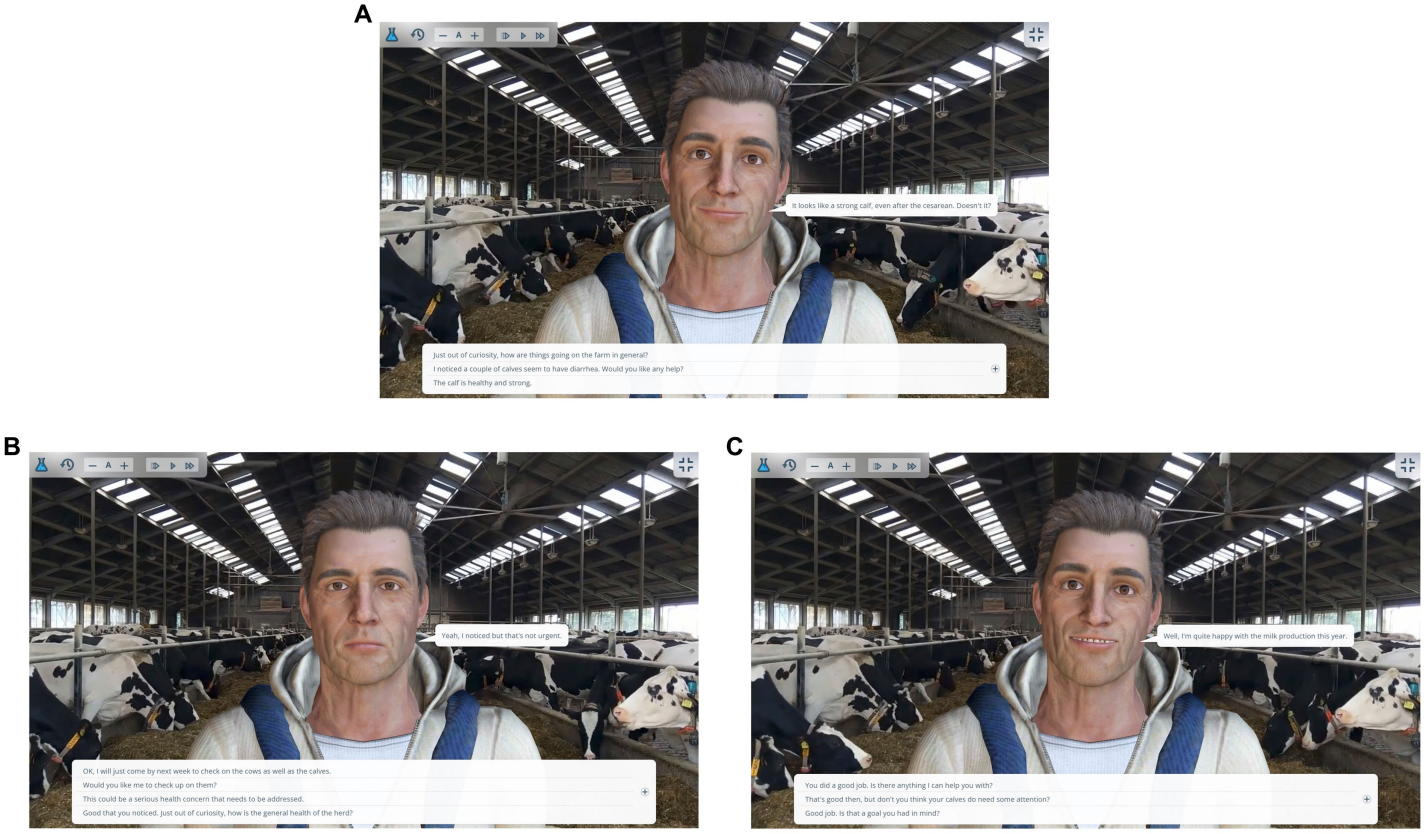
**(A)** Screenshot of a node in a VDT scenario. The player can click one of the answer possibilities or click the “+” on the right to suggest alternatives. In **(B,C)**, results of choosing the first or second options are demonstrated. **(B)** Screenshot of a node in a VDT scenario. The avatar’s response after choosing the option “I noticed a couple of calves have diarrhea. Would you like any help?’ in **(A)**. **(C)** Screenshot of a node in a VDT scenario. The avatar’s response after choosing the option “Just out of curiosity, how are things going on the farm in general?” in **(A)**.

The VDT offers a platform for veterinary students and professionals to engage in simulated conversations with a virtual client in a veterinary setting (Figure 1). A demonstration account is available at https://en.dialoguetrainer.app/join/JVABAQAX. In this study, participants in the training intervention completed the simulation “First contact.” Simulations were played by veterinary practitioners (the player) who engaged with a virtual dairy farmer (the client). The VDT was designed to make it easily accessible, intuitive, and self-explanatory. No previous experience with the tool was needed. During a simulated farm visit, the virtual farmer tells (through a speech bubble and voice over) about a situation or issue on their farm in a way that warrants a reaction from the playing veterinarian (Figure 2A).

Then, the player has the possibility to click on one of multiple predefined player choices, called a “node” in the scenario. The player chooses the option that is closest to how they would intend to respond in a similar situation. The player’s response elicits a reaction from the virtual farmer, either verbally or non-verbally (change in posture and/or a thought in a thought balloon) and directs the conversation further (Figures 2B,C). At each node in the conversation, the intention of the player’s choices influences continuation of the conversation in a positive, neutral, or negative direction. Depending on choices, the conversation ends when the player has given the farmer final advice and concludes the visit, or, in case they have upset the farmer, by the farmer asking them to leave the farm. After completing the game, the player is presented with a total score and sub scores for three parameters (see “scoring of scenarios”) and an overview of all steps in the conversation, with feedback for every step.

### 2.3. Development of VDT communication scenarios

**Figure 3:**
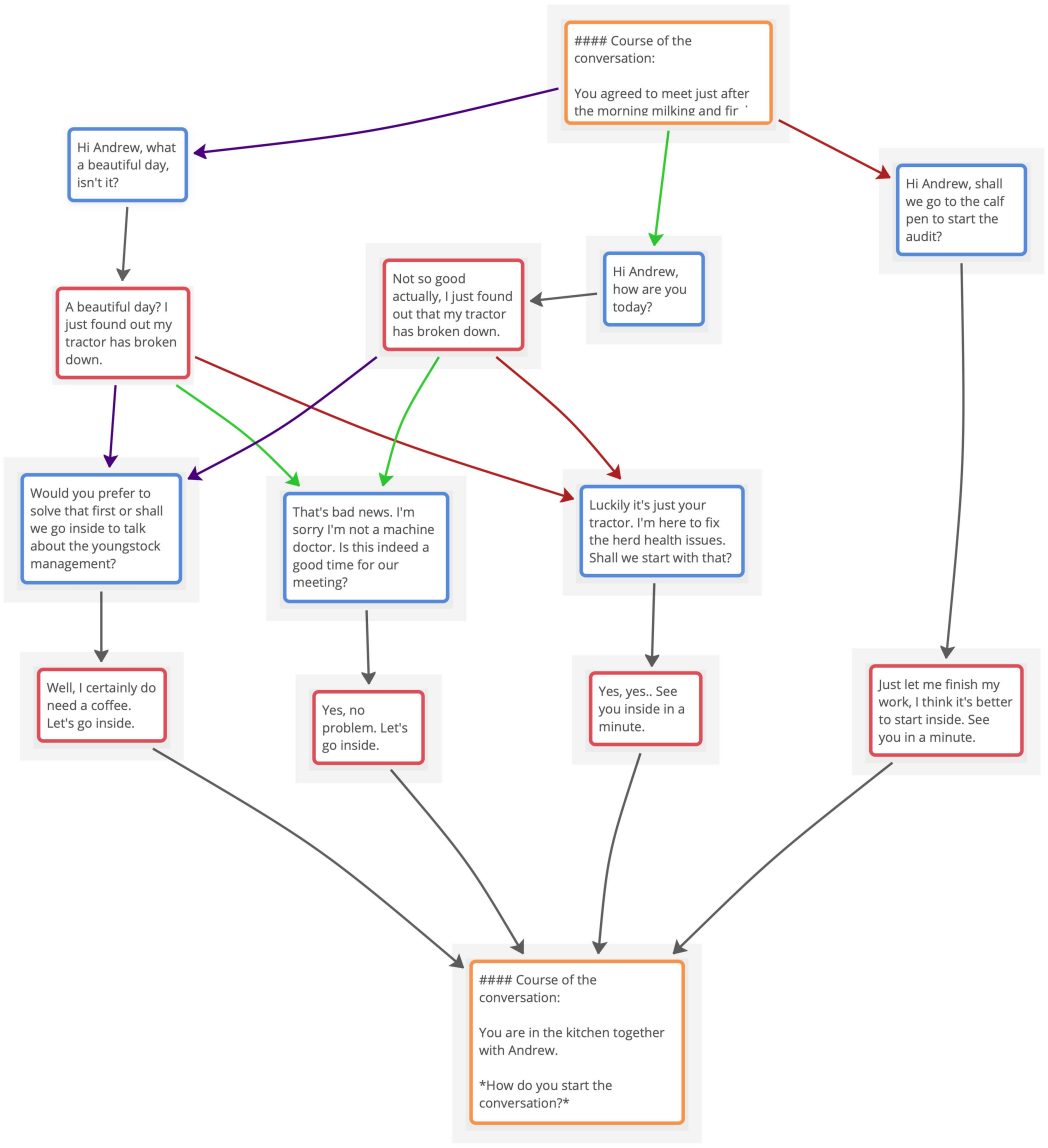
Screenshot of a scenario decision tree. This is an example scenario, it is not used in this study. Orange box: information or knowledge transfer to the player, e.g., providing background of the conversation, staging the scenario. Blue box: answer possibilities for the player of the scenario. Red box: response of the avatar. Green arrow: best communication practice. Purple arrow: sub-assertive/less effective answer. Red arrow: too direct, dominant, or even (passive) aggressive answer.

Scenarios were developed according to a five-step process. First, the situation for which a scenario would be developed was determined and learning objectives, target audience and content of situation-specific conversations explored. Consequently, in collaboration with communication experts, farmers and veterinary practitioners, the best communication practices for each situation were determined based on behavioral theory (32, 33), motivational interviewing methodology, and Calgary-Cambridge guidelines (12, 34). In the third step, “Do’s and don’ts” were determined for the specific situation of the simulated conversation, and aligned with previously established learning objectives, e.g., “Building the client relationship.” Fourth, the scenario was written and converted into a simulated conversation, i.e., the simulation (Figure 3). Multiple answer possibilities (i.e., player statements) for each node enabled participants to choose an option matching their intention in the node, e.g., showing empathy, being time efficient. Additionally, the developer enhanced the character, complexity, and depth of the virtual farmer/client, e.g., by adding certain body postures and thought balloons.

### 2.4. Scoring of simulations

Learning goals in the simulations used in this study were related to “Building the relationship,” “Clarifying needs,” and “Showing added value,” the three domains included in training intervention (see “training intervention”). Learning objectives in a simulation are referred to as parameters, which are encoded as integers (35). Underlying communication skills that are represented in statements of the player contribute to an incremental score on parameters, e.g., when the player selects an option with an open-ended question that seeks to understand the needs of the virtual farmer, the parameter “Clarifying needs” will change by +1 (Table 1). Additionally, the statement can have an effect on the virtual character, e.g., “Relief,” and provide textual feedback for the player. Ultimately, a player’s score on a parameter is the percentage of the maximum possible obtainable score for that parameter. Scores on the parameters “Identifying needs,” “Relationship building,” and “Showing added value” were averaged to a total score “Bovine Preventive Consultant” on a 0–100% scale.

**Table 1:** Examples of underlying communication skills contributing to parameters (12).

	Parameter		
	Building the relationship	Clarifying needs	Showing added value
Underlying communication skills	Establishing initial rapport, demonstrating empathy, involving the avatar, affirming the avatar	Asking open-ended and follow-up questions, reflective listening statements, perceiving cues	Offering veterinary services without pushing, establishing follow-up plan and/or appointments, explaining costs

### 2.5. Training intervention

Communication training focused on communication skills needed to be a successful veterinary herd health consultant and was based on Motivational Interviewing methodology (34) and Calgary-Cambridge guidelines (12). The main language during training intervention was English. During training, the background and development of the VDT were introduced, communication simulations played, general feedback on previously obtained scores given, and time allocated to repeat simulations to enhance learning. Players were encouraged to not solely try to obtain a high score, but afterwards also ‘play around’ to enhance learning about undesirable ways of communication.

Finnish participants were invited to create a user profile for the VDT and to play the communication simulations provided before the training and at set moments during training. Swedish participants created a user profile at the beginning of the training.

Training for the Finnish participants lasted 2 d, with five simulations with increasing difficulty provided to participants (Figure 1). In the first simulation, the player initiated the conversation regarding herd health with the virtual farmer; the second scenario depicted a herd health visit conversation; the third scenario addressed the follow-up of a herd health conversation; and in the fourth scenario the player had a conversation with a “hard-to-reach” farmer, i.e. a farmer that is more resistant to change. The fifth and final scenario brought together all previous simulations, enabling the player to use all experiences.

Training duration for the Swedish participants was 4 h and covered similar topics, albeit not as in-depth as the 2-d training. Due to limited time, these participants took part in the first simulation only. As the first simulation was played by both groups, this manuscript will focus on scores of that specific simulation.

### 2.6. Net promoter score

Participants were given opportunities to provide feedback by means of the Net Promotor Score (NPS), an index ranging from −100 to +100 measuring participants’ willingness to recommend the course to others (36). For Finnish participants this was possible after Day 1 and completion of training. For Swedish participants this was possible right after completion. The NPS was assessed by asking participants the following question: “On a scale from 0 to 10, how likely are you to recommend this training to others?” Responses were grouped; promoters were the participants who gave a score of 9 or 10, and detractors were those who indicated 6 or lower. Finally, responses were compiled and percentage of detractors was subtracted from percentage of promoters (36).

### 2.7. Questionnaire

Participants were asked to provide general demographic data as well as information specific to their veterinary practice and prior experience with communication training (Appendix 1).

### 2.8. Data analyses

Data were compiled in Microsoft Excel Version 16.69 (Microsoft Corp., Redmond, WA, United States), which was also used for descriptive analyses. Statistical analyses were performed with Rstudio 2022.07.2, build 576 (PBC, Boston, MA, United States, http://www.rstudio.com/). We explored attained scores and number of attempts by participating veterinarians by creating tables and descriptive statistics like mean and median. Scores of participants at consecutive attempts were compared using paired Student’s *t*-tests (H_0_: mean difference = 0). Separate *t*-tests were conducted for data from Finnish and data from Swedish participants. Statistical significance was declared at a *p-*value < 0.05.

## 3. Results

### 3.1. Participants

In the Finnish training, 35 of the 51 initial invitees actively participated in training, of which 24 completed the first VDT scenario multiple times. The Swedish training had 25 participants taking part in the online communication simulations; 21 participants completed the communication simulation at least twice and met inclusion criteria for this study. Reasons for not playing the simulation, or not playing repeatedly, included lack of a device, lack of internet connection, and time constraints.

Demographic characteristics of participants are presented in Table 2. Twelve (50%) Finnish and 18 (75%) Swedish participants worked in bovine medicine for the majority of their time (>50% of the time/week).

**Table 2:** Demographic characteristics (No, %) of veterinarians participating in training in Finland or Sweden, respectively.

	Finland	Sweden
**Gender** ^a^		
Female	23 (96)	17 (81)
Male	1 (4)	4 (19)
**Employment** ^a^		
Clinic, Owner	6 (25)	0 (0)
Clinic, Associate	4 (17)	11 (52)
Clinic, Locum	3 (13)	6 (29)
Government	9 (38)	1 (5)
Industry	1 (4)	2 (10)
Teaching	1 (4)	1 (5)
**Clinic size** ^b^		
Full-time veterinarians	3, 3 (0–8)	14, 1 (0–85)
Part-time veterinarians	2, 1 (0–20)	4, 1 (0–33)
Age^b^ (y)	42, 40 (30–66)	43, 41 (30–63)
Duration of experience in veterinary practice^b^ (y)	13, 11 (1–32)	12, 11 (1–35)
**Practicing in** ^a^		
Finland	24 (100)	0 (0)
Estonia	1 (4)	0 (0)
Sweden	1 (4)	20 (95)
Other	0 (0)	1 (5)
**Work activities** ^b^**, estimated proportion of work hours per week spent working in (%)**		
Bovine	55, 55 (0–100)	70, 100 (0–100)
Small animal	28, 25 (0–100)	2, 0 (0–30)
Equine	5, 0 (0–33)	9, 0 (0–70)
Other	13, 0 (0–100)	19, 0 (0–100)
**Working hours** ^b^**(h/week)**		
Full-time working participants	47, 47 (36–71), *n* = 14	42, 40 (40–50), *n* = 13
Part-time working participants	29, 32 (5–40), *n* = 9 (1 missing)	32, 32 (30–35), *n* = 8
HHPM farm visits^c^ (no/month)	10, 6 (1–30)	9, 5 (0–40)
Previously participated in communication training^a^	7 (29)	14 (67)

a Values are no. (%). Percentages are based on total number of 24 (Finland) and 21 (Sweden) participating veterinarians. Percentages may not add up to 100% due to rounding.

b Values are mean, median (minimum–maximum).

c Herd health and production management.

Seven (29%) Finnish and 14 (67%) Swedish participants received some form of veterinary communication training before participating in the study. Their experiences ranged from a few hours to multiple (multi-day) courses. Participant’s mean scores, stratified by previous communication skills training experience, are in Table 3.

**Table 3:** Average Veterinary DialogueTrainer scores of participants on parameters that were the foundation of veterinary communication training; “Clarifying needs,” “Relationship building,” and “Showing added value.”

		Finland (*n* = 24)				Sweden (*n* = 21)			
		Clarifying needs	Relationship building	Showing added value	Total score	Clarifying needs	Relationship building	Showing added value	Total score
Did not previously receive communication skills training	1st	18 (7)	11 (7)	2 (6)	10 (7)	52 (35)	50 (37)	35 (47)	46 (39)
	2nd	46 (31)	44 (32)	12 (14)	34 (26)	41 (31)	40 (32)	10 (12)	30 (24)
	best	69 (39)	66 (40)	62 (49)	66 (43)	91 (10)	88 (13)	80 (40)	86 (20)
Did previously receive communication skills training	1st	14 (5)	10 (8)	0 (0)	8 (4)	53 (21)	57 (19)	22 (16)	44 (17)
	2nd	52 (38)	53 (37)	30 (48)	45 (40)	61 (30)	68 (27)	27 (28)	52 (27)
	best	89 (17)	88 (17)	70 (38)	82 (26)	87 (20)	91 (9)	78 (35)	85 (21)

Stratified by previous experience with communication skills training. Finnish participants reached their best total score in on average 3.5 (SD ± 2.1) times. Swedish participants reached their best total score in on average 3.1 (SD ± 1.1) times. Mean score for first attempt, second and best attempts in % (±SD).

### 3.2. Number of attempts

The range of attempts varied greatly, with 2 veterinarians playing the simulated conversation 11 times (Table 4). Most participants reached a total score >80% and 7 (Finland) and 8 (Sweden) players reached a 100% score. Most players stopped playing the simulation after obtaining their high score. However, 15 participants (6 Finnish, 9 Swedish) continued playing for on average 2 additional attempts (SD ± 1.3) after obtaining the maximum score.

**Table 4:** Attempted plays of the VDT simulation.

	Finland, *n* = 24	Sweden, *n* = 21
Mean number per player	4.1 (2.7)	3.9 (1.3)
Reaching a total score >80%	4.0 (1.6), *n* = 17	2.8 (1.0), *n* = 16
Reaching score 100%	5.3 (1.8), *n* = 7	3.4 (0.5), *n* = 8

Number of attempts (±SD).

### 3.3. Obtained scores

At the first attempt of playing the scenario 39 (87%) participants had a total score <50%, most (*n* = 34) increased their initial score at the second time playing the simulation. Almost all (*n* = 41, 91%) participants increased their scores after playing multiple times; the others achieved their highest score at the first attempt and thereafter did not get a higher score.

**Figure 4:**
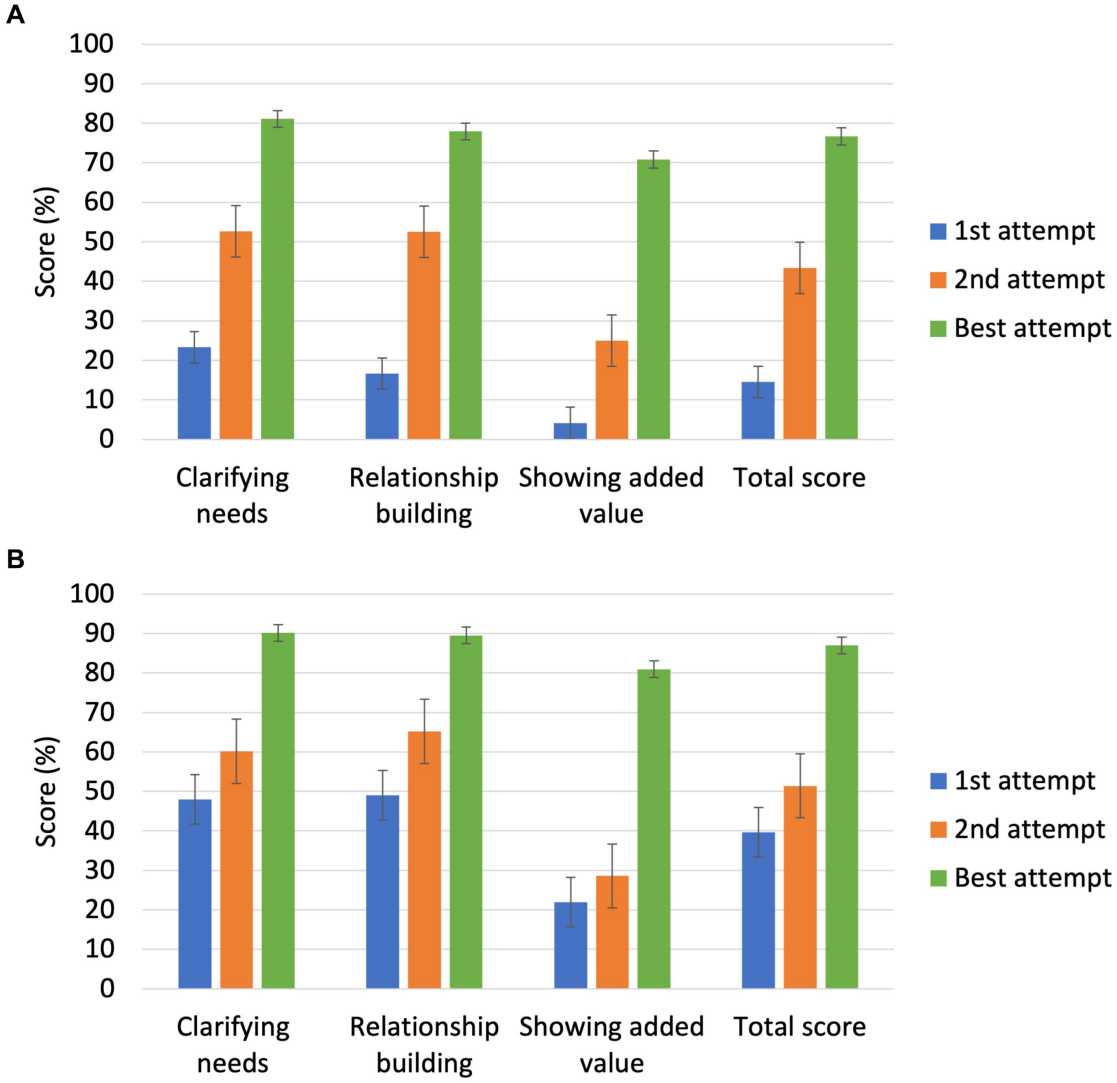
**(A)** Average scores (%) of participants’ first, second, and best attempts on the simulation for communication skills parameters. Finnish participants reached their best total score in on average 3.5 (SD ± 2.1) times. Finnish participants, *N* = 24. **(B)** Average scores (%) of participants’ first, second, and best attempts on the simulation for communication skills parameters. Swedish participants reached their best total score in on average 3.1 (SD ± 1.1) times. Swedish participants, *N* = 21.

Mean scores for the Finnish participants between the first and the second attempt increased on “Clarifying needs,” “Relationship Building,” “Showing added value,” and total score (Figure 4A; *p* < 0.05). However, scores of Swedish participants did not increase between first and second attempts (Figure 4B). For both Finnish and Swedish participants, mean scores on all parameters increased between the first and the best attempt, with participants reaching a total high score after playing the simulation on average 3.3 (SD ± 1.7) times.

### 3.4. User feedback

The NPS question was answered by 35 Finnish training participants on training Day 1, the NPS for the first day was +89 (four times an 8, 10 times a 9, and 21 times a 10). The second day the question was answered by 32 participants and received a score of +88 (one 7, 3 times 8, 11 times a 9, and 17 times a 10). Twelve Swedish participants answered the question, the NPS was +83 (2 times an 8, 4 times a 9, and 6 times a 10).

## 4. Discussion

This study aimed to: introduce and evaluate the use of the Veterinary DialogueTrainer in post graduate communication training; determine whether the VDT improved virtually assessed communication skills scores; and report on the willingness of participants to recommend this online role-playing tool to other veterinarians.

Compared to the 2018 report of the Federation of Veterinarians of Europe (37) in which Finland and Sweden had a female veterinary population of 89 and 82%, respectively, the gender distribution of participating veterinarians was skewed more toward women (96 and 81%). Participating food animal veterinary practitioners from Finland were of similar age [in Norring et al. (38) mean age was 42 ± 10 y, data not available for Sweden] and practice experience was similar for both Finnish and Swedish participants [(38), 14 ± 10 y; in Reijula (39) 39.1% had >15 y experience]. Workload (excluding on-call shifts) in our study was slightly less (39.7 h/week) than reported by Reijula et al. (39) who mentioned 41.7 h/week for women and 44.1 h/week for men. However, when stratified into whether participants work on a full-or part-time, participants worked ~45 or 30 h/wk., respectively.

There was no apparent difference between scores of participants who had or had not previously received communication skills training. Due to a lack of standardization in previous training experiences, it was not possible to comment on effectiveness of that training. It was noteworthy that Finnish participants who previously received communication skills training increased their scores to a greater extent compared to those who had not.

In this study the VDT is used as part of a group training to facilitate a blended learning experience. An advantage of it being part of a training is debriefing and reflection with facilitators and fellow participants after engaging with the simulation. This way, the learning experience is deepened, and retention might be improved. In a different setting the VDT can also be used as an individual stand-alone e-learning. When individually playing the simulation, the participants receive feedback on their scores and can learn from mistakes by playing multiple times. The use of the tool in different settings is currently subject of further study.

Most participants scored <50% at the first attempt playing the Veterinary DialogueTrainer simulation, and all were able to improve their score. This indicates that choosing the best practice answers in the simulations, leading to a 100% score, was difficult for most participants on their first attempt. The results also indicate that participants were able to choose more effective options to communicate with the avatar leading to increase of scores.

We choose 50% for this manuscript as it is widely understood as pass/fail in assessments. Whether this is an adequate interpretation of the scores that participants get for playing the simulations, is subject to further study.

This study was the first exploration whether the tool was not too easy, e.g., most of the participants scoring very high on the first try of the simulation, not too hard, e.g., participants were able to obtain the high score, and whether participants were able to improve their scores from baseline. Additionally, this is a first exploration of participants’ skill level in determining what the best communication practice in a simulation might be and whether that level seems to increase with training. What score or what increase in score is “enough,” and how that translates to real life consultations is subject to further study.

Scores on various parameters of the communication simulation increased from the first attempt to the second for the Finnish veterinarians, whereas scores increased from the first attempt to the best for both Finnish and Swedish veterinarians. There was an apparent difference between Finnish and Swedish veterinarians, as the latter had higher scores for both first attempt and for best scores. That veterinary communication is widely studied in Sweden (19, 40) may have enhanced understanding of veterinary communication skills in Swedish veterinarians. Additionally, selection bias may have occurred due to language of training being English. The participants typically communicate with their clients in the client’s local language, i.e., Finnish, Swedish and/or Estonian. Simulations included in the tool can be translated to any language. This might improve the usefulness and adoption of the tool as a possible language barrier is diminished. Participants generally had good functional English and voluntarily joined the training, which may have influenced their performance. Cultural differences may also have influenced willingness to participate and perform. More research is needed to study the effect of the language of the tool on the participation and the performance.

Interestingly, participants scored lower on the parameter “Showing added value” than on “Clarifying needs,” and “Relationship building.” This might be a result of the flow of the scenario, where opportunities to score on this parameter occur later in the simulation than the other two. If the player “upset” the virtual farmer and the conversation was terminated prematurely by the avatar, this decreased opportunities to score on “Showing added value.”

Additionally, that participants played the simulation multiple times, even after having obtained their highest score, demonstrated they were motivated to engage with the tool and that a barrier to engage in role-play may have been lifted (25). This sentiment was supported by the Net Promoter Score scores of +89, +88 (Finland) and +83 (Sweden) indicating participants were likely to recommend this training to other veterinarians.

Based on current results, the Veterinary DialogueTrainer could satisfy the learning conditions stated by Ericsson’s model on expert performance (41). This model articulates that deliberate practice in specified learning circumstances is the only way to achieve expert levels of performance. Learning conditions that should be met are “(a) performing learning tasks with well-defined goals; (b) motivated to improve; (c) learning tasks of short duration with opportunities of immediate feedback, reflection and corrections; and (d) ample opportunities for repetition, gradual refinements and practice in challenging situations (41).” After restricted training, i.e., training where these conditions are not met, an individuals’ performance merely adapts to typical situational demands on a satisfactory level; more experience will therefore not result in improved behavior and expert performance. The VDT could have an important role in beforementioned deliberate practice and meeting learning conditions. VDT simulations can be designed for specific and clearly defined learning objectives (Condition a). In addition, Condition b can be met, demonstrated by players playing multiple times and continuing to play after having reached their high scores. This provides learners with an opportunity of repeated practice of short learning tasks, while directly receiving feedback and providing a chance for reflection and correction (condition c). Additionally, as it is an online tool that can contain multiple scenarios of ascending difficulty, it offers the possibility of repeated learning experiences and practice of challenging situations (condition d).

Some veterinary work (40, 42) demonstrated that after a short communication training of 4–5 h in the UK or a 6-month training program in Sweden in motivational interviewing (a communication methodology where practitioners are trained to evoke motivation of their clients for a specific behavioral change), even though communication competencies generally improved, only 21 and 6% of participants, respectively, were able to reach “fair competency” for these skills, emphasizing the importance of deliberate practice over prolonged time to master a skill. The VDT could have a role in deliberately practicing communication skills as simulations are continuously available.

As training is increasingly being offered online, an advantage of the VDT is that it provides the veterinary field a scenario building tool capable to build simulations for a vast variety of settings. The VDT is not only suitable to simulate conversations between vets and their clients but allows simulations of all types of conversations, as a wide range of avatars, backgrounds, and emotions is available. Once a simulation is built, it can easily be translated into any language, making it easily scalable for larger and diverse audiences.

Communication between veterinarian and client exists of verbal and non-verbal transfer of messages. In the VDT, participants are limited to communication through pre-determined written answers. Even though choosing between answers might increase the participant’s understanding of ways to respond in certain situations and therefore adds to the learning experience, these pre-determined answers also limit the response options. So, a gap is present between the tool and real-life conversations, in which a person can use a variety of answers, tone of voice, facial expression etc. in their communication. Work is ongoing to narrow this gap. For example, since delivering the training, the VDT has been developed further to enable open text responses of participants, e.g., typing or audio recording their response.

Whether increased communication scores measured by the VDT translate into increased communication skills in veterinary practice requires further study. Nevertheless, in a systematic review by Boyle et al. (43), studies examining serious games that focused on knowledge or skills acquisition, playing such games increased performance compared to control groups. Furthermore, Schönbrodt et al. (44) demonstrated connections between behaviors toward a virtual spouse in a computer-generated world and intimacy motives and satisfaction with real-world relationships, implying some transmission occurred between real and virtual worlds. Communication skills scores as measured by the VDT increased on the levels of clarifying needs, relationship building, showing added value, and the total score. Research currently underway will explore whether communication scores in VDT are linked to real world behavior by participating veterinarians, providing an exciting opportunity to add virtual role-play to the toolbox of veterinary clinical communication educators.

After playing a scenario, the participant receives specific feedback for each node in the simulated conversation. This feedback is highly useful in both “well-played” and “poorly played” simulations (i.e., for high and for low scores), as it provides advice on what to do and what to avoid in the specific context of the scenario. Therefore, players of the simulations are encouraged to aspire to get both a 100% score and a low score, e.g., by making the farmer “angry,” to deepen their learning in both situations. However, this results in the situation that no assumptions can be made about participants’ motivations in playing when they have a low score, i.e., are they actively trying to obtain a poor score for the feedback or for fun or were they unable to get a high score. Therefore, no conclusions can be made on whether 0% scores were deliberate, i.e., to “upset” the virtual farmer to learn from the feedback, or not. In the future, this challenge could easily be mitigated by instructing players to aim for improving their scores up to a high score first, before deliberately “upsetting” the virtual farmer.

## 5. Conclusion

This study demonstrated the VDT to be a promising tool for use in veterinary communication training. Participants appreciated the learning experience and increased their scores in multiple attempts. Irrespective of challenges faced, we predict a clear benefit of using the VDT in teaching veterinary communication competencies.

## Data availability statement

The raw data supporting the conclusions of this article will be made available by the authors, without undue reservation.

## Ethics statement

The studies involving human participants were reviewed and approved by University of Calgary’s Research Ethics board. The patients/participants provided their written informed consent to participate in this study.

## Author contributions

LD, CR, and HB conceived of the presented idea. JJ and GV organized and facilitated the training and data collection and were in charge of planning. LD and JJ collected the data. TP supported LD in extracting the data from the VDT platform. LD processed the data, performed the analysis, drafted the manuscript, and designed the figures. CR and HB aided in interpreting the results and supervised the project. LD, JJ, and TP wrote the manuscript with input from all other authors. All authors contributed to the article and approved the submitted version.

## Funding

This study was supported by the Industrial Research Chair in Infectious Diseases of Dairy Cattle, funded by Canada’s Natural Sciences and Engineering Research Council (NSERC) Industrial Research Chair Program (Ottawa, ON, Canada), with industry contributions from Alberta Milk (Edmonton, AB, Canada), the Dairy Farmers of Canada (Ottawa, ON, Canada), Westgen Endowment Fund (Milner, BC, Canada), the BC Dairy Association (Burnaby, BC, Canada), Canadian Dairy Network (Guelph, ON, Canada), CanWest DHI (Guelph, ON, Canada), SaskMilk (Regina, SK, Canada), Dairy Farmers of Manitoba (Winnipeg, MB, Canada), and MSD Animal Health (Kirkland, QC, Canada).

## Conflict of interest

St. Anna Advies B.V. (www.anna-advies.com, Beuningen, The Netherlands) holds a license to use the DialogueTrainer platform in a veterinary setting and developed the simulations used in this study. Employees of St. Anna Advies had no input on the study design or which results are presented in this manuscript. GV is an employee of MSD Animal Health. He had no input on the study design nor which results were presented in this manuscript.

The remaining authors declare that the research was conducted in the absence of any commercial or financial relationships that could be construed as a potential conflict of interest.

## Publisher’s note

All claims expressed in this article are solely those of the authors and do not necessarily represent those of their affiliated organizations, or those of the publisher, the editors and the reviewers. Any product that may be evaluated in this article, or claim that may be made by its manufacturer, is not guaranteed or endorsed by the publisher.

## Appendix 1. Questionnaire participants

1. What is your year of birth? ____.

2. What is your gender?

- Male.
- Female.
- Non-binary.
- None of these applies to me. I prefer to identify as …

3. What is the number of years you have been practicing as a veterinarian in veterinary clinical practice?

4. In which country do you work as a veterinarian?

5. What is your main type of work? Indicate the percentage of time you spend working in the following:

- % bovine.
- % small animal.
- % equine.
- % other, please specify ……

6. How many herd health visits do you conduct on average each month?

7. What is your position in your veterinary clinic.

- Owner.
- Associate.
- Locum.
- Other, please specify ……

8. Are you working full time or part time?

Part time, … hours a week, a full-time week in your clinic being … hours a week.

Full time, a full-time week in your clinic being … hours a week.

9. How many veterinarians are working in your clinic?

…full-time veterinarians, … part-time veterinarians.

10. Did you participate in any communication training before? This may include training during your veterinary education as well as continuing communication training.

- Yes
- No

If you answered yes, please describe the length and character of the communication training.
